# Mitochondrial superoxide dismutase overexpression and low oxygen conditioning hormesis improve the performance of irradiated sterile males

**DOI:** 10.1038/s41598-021-99594-1

**Published:** 2021-10-12

**Authors:** Vanessa S. Dias, Carlos Cáceres, Andrew G. Parker, Rui Pereira, Güler Demirbas-Uzel, Adly M. M. Abd-Alla, Nicholas M. Teets, Marc F. Schetelig, Alfred M. Handler, Daniel A. Hahn

**Affiliations:** 1 Insect Pest Control Subprogramme, Joint Food and Agriculture Organization (FAO)/International Atomic Energy Agency (IAEA) Centre of Nuclear Techniques in Food and Agriculture, 1400 Vienna, Austria; 2 Department of Entomology and Nematology, University of Florida, Gainesville, 32611 USA; 3 Department of Entomology, University of Kentucky, Lexington, 40546 USA; 4 Department of Insect Biotechnology in Plant Protection, Justus-Liebig-University Gießen, 35394 Gießen, Germany; 5 Center for Medical, Agricultural and Veterinary Entomology, USDA/ARS, Gainesville, 32608 USA

**Keywords:** Biotechnology, Ecology, Evolution, Genetics, Zoology

## Abstract

The Sterile Insect Technique (SIT) is a successful autocidal control method that uses ionizing radiation to sterilize insects. However, irradiation in normal atmospheric conditions can be damaging for males, because irradiation generates substantial biological oxidative stress that, combined with domestication and mass-rearing conditions, may reduce sterile male sexual competitiveness and quality. In this study, biological oxidative stress and antioxidant capacity were experimentally manipulated in *Anastrepha suspensa* using a combination of low-oxygen conditions and transgenic overexpression of mitochondrial superoxide dismutase (SOD2) to evaluate their role in the sexual behavior and quality of irradiated males. Our results showed that SOD2 overexpression enhances irradiated insect quality and improves male competitiveness in leks. However, the improvements in mating performance were modest, as normoxia-irradiated SOD2 males exhibited only a 22% improvement in mating success compared to normoxia-irradiated wild type males. Additionally, SOD2 overexpression did not synergistically improve the mating success of males irradiated in either hypoxia or severe hypoxia. Short-term hypoxic and severe-hypoxic conditioning hormesis, per se, increased antioxidant capacity and enhanced sexual competitiveness of irradiated males relative to non-irradiated males in leks. Our study provides valuable new information that antioxidant enzymes, particularly SOD2, have potential to improve the quality and lekking performance of sterile males used in SIT programs.

## Introduction

Insects tolerate high doses of radiation relative to other taxa, a feature due mainly to the low levels of cellular division occurring in advanced stages of the insect life cycle^1,2^, with dipteran cells being three to nine times more radiotolerant than mammalian cells^3^. The same tolerance does not apply for undifferentiated and highly radiosensitive insect cells, particularly germ cells, such as spermatogonia, spermatocytes, and spermatozoa^4^. The direct effect of ionizing radiation leads to severe and irreversible double-stranded DNA damage (dominant lethal mutations) in germ cells that causes insect sterilization^5,6^, while the functioning of somatic cells that do not divide is less affected. This is why insects sterilized with ionizing radiation for SIT are typically treated in either the pharate-adult stage just before adult emergence when > 95% of morphogenesis is complete, or as fully emerged adults^2^. Only a few types of somatic cells can also divide during the adult stage of insects and, consequently, may be seriously affected by irradiation. For instance, irradiation of pharate adult fruit flies resulted in damage to midgut cells and the stem cells that replace them, evidenced by presence of distorted nuclei and mitochondrial deformation^7^. This difference in radiosensitivity between germline (dividing cells) and somatic cells (post-mitotic cells) make insects good candidates for the sterile insect technique (SIT), a pest control method based on the sterilization of insects released in the field to control a target pest^8^.

Although most somatic cells of irradiated males used in SIT programs remain functional, ionizing radiation damages these cells. The primary indirect effect of ionizing radiation to living organisms is the generation of reactive oxygen species (ROS) through the radiolysis of water, particularly the formation of hydroxyl radicals and hydrogen peroxide, which can cause damage to both somatic and germ cells^9–11^. Excessive amounts of ROS that overwhelm cellular defenses result in oxidative damage to DNA, proteins, and lipids that, ultimately, affect organismal performance^12,13^. Fortunately, cells have molecular machinery that can mitigate the harmful effects of ROS, specifically antioxidant enzymes, small molecule antioxidants (e.g., glutathione), and chaperones that act together with diet-derived antioxidants (e.g., carotenoid pigments) to reduce and/or repair cellular damage caused by oxidative stress^12,14–16^. These antioxidants are ubiquitous in animals and minimize the detrimental effects of oxidative stress not only in insects^16^, but also in birds, lizards, fish, and mammals^12,14,15^. Because of their widespread use in SIT programs, the whole-organism effects of radiation exposure, associated with increased levels of ROS and consequent oxidative damage, have been well characterized in several tephritid fruit flies^3,17–25^. For instance, *Ceratitis capitata* males irradiated under normoxia showed a nearly two-fold reduction in mating performance compared to non-irradiated males^24^. Many tephritids use an energetically costly lek-based mating system^39^, and this mating strategy is expected to be difficult to perform under periods of stress. The applied importance of tephritid flies, coupled with their tractability in the lab, make these insects good systems for investigating the relationship between cellular oxidative balance and fitness.

Insects used in SIT programs that are irradiated with gamma and X-rays exhibit significant decreases in quality and sexual competitiveness when irradiated in normal air, but irradiation is less deleterious when pupae are held in low-oxygen conditions^26,27^. Greater radiosensitivity in the presence of oxygen, a phenomenon called the oxygen effect, has been well known since the 1940s^28^. In recognition of this oxygen effect, for decades operational tephritid fruit fly SIT programs have irradiated insects in modified atmospheres with either low levels or no oxygen to reduce radiosensitivity and mitigate the damaging effects of ionizing radiation exposure^2,29,30^. The protective effects of low-oxygen on tephritid fruit flies in the context of SIT has been shown by many studies going back to the 1970s^18,31–36^. However, this large body of literature is lacking in molecular mechanistic evidence for the causes of this oxygen effect. Currently, it is recognized that damage in mitochondria, instead of purely fixed damage to nuclear DNA, as predicted by the oxygen fixation hypothesis, accounts for most cellular radiosensitivity observed when irradiation occurs in the presence of oxygen^11^. Our long-term goal is to improve the sterilization process such that off-target damage to somatic cells is reduced without compromising the damage to germline genomic DNA required for sterilization. These improvements will enhance the performance of sterile males and allow them to maintain competitiveness with wild males, which is critical for effective SIT^2,4–8^. Knowledge of the molecular mechanistic basis of radioprotection by low-oxygen conditioning is thus critical for designing manipulations and treatments to improve the performance of sterile males used in SIT programs.

One possible molecular mechanism explaining the radioprotective effect of anoxia (lack of oxygen) conditioning was recently proposed in the Caribbean fruit fly *Anastrepha suspensa* (Loew)^37^. Briefly, irradiation of tephritid fruit flies pre-conditioned in anoxia resulted in greater total antioxidant capacity, particularly mitochondrial superoxide dismutase (SOD2) activity, less oxidative damage, and better sexual performance compared to unconditioned irradiated males. Later, the same authors showed that the hormetic benefits of anoxia conditioning were also carried into old age by reducing oxidative damage and increasing longevity and extending the duration of mating competitiveness in those insects^38^. Recently, we demonstrated a specific protective effect for SOD2 by overexpressing SOD2 in *A. suspensa* and showing that it reduced oxidative damage to lipids, improved mating performance, and preserved locomotor activity in irradiated insects^40^.

In this study, biological oxidative stress^41^ and antioxidant capacity were experimentally manipulated using a transgenic line of *A. suspensa* that overexpresses SOD2 (line SOD2 5.2 from reference^40^) to directly evaluate the role played by this cellular antioxidant enzyme in the sexual behavior and quality of irradiated males. Specifically, we tested the degree to which enhancing antioxidant capacity in transgenic flies combined with low-oxygen atmospheres could reduce biological oxidative stress (an adverse side effect of ionizing radiation), increase male mating success, and improve insect quality under radiation (severe oxidative stress). We hypothesize that males overexpressing SOD2 are more sexually competitive than wild-type (WT) males that do not overexpress SOD2 under irradiation in normoxia (Nx, normal air) in field-relevant settings. Additionally, we hypothesize that SOD2 overexpression interacts synergistically with short-term hypoxic (Hx) or severe hypoxic (SHx) conditioning hormesis to improve mating success in field cages and overall quality of *A. suspensa* males.

Through a series of experiments, we evaluated multiple biological parameters to test our hypotheses. First, we measured the total antioxidant capacity of gamma-irradiated and non-irradiated WT and SOD2 5.2 males treated under different atmospheric conditions. Second, we evaluated the sexual competitiveness of SOD2 5.2 and WT flies in field cages that permit formation of leks, which is a substantial extension of our previous work in small cages^40^ that only allow pairs of competing males. Third, assuming that lek territories with intense male-male competition are more frequently occupied by high-quality individuals, the locations where the males mated within the tree canopy were evaluated to understand the extent to which SOD2 overexpression protects the performance of irradiated males in leks. Finally, we assessed quality control parameters, such as emergence, deformation, flight ability, and sterility of irradiated and non-irradiated insects, treated or not with hypoxia and severe hypoxia. The results from this study can be used to assess the degree to which the sexual advantage observed in transgenic males tested in a previous study^40^ extends from small laboratory cages to larger cages in semi-natural conditions^29^. Also, these experiments are important for evaluating whether SOD2 overexpression can be combined with hormetic conditioning by hypoxia to further improve the sexual performance of irradiated *A. suspensa* males.

## Results

### Total antioxidant capacity

**Figure 1:**
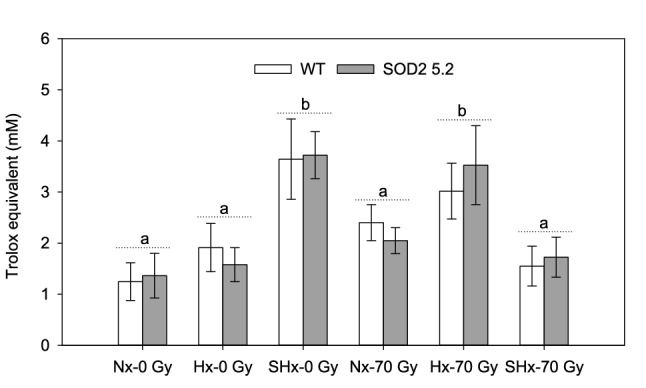
Total antioxidant capacity measured in Trolox equivalents of irradiated (70 Gy) and non-irradiated (0 Gy) WT and SOD2 5.2 males under normoxia (Nx), hypoxia (Hx), and severe hypoxia (SHx) atmospheric conditions. Bars represent means with standard errors of Trolox equivalents measured in samples with four non-irradiated and irradiated males treated or not with low-oxygen atmospheres. Bars followed by different letters are significantly different from each other (LS-means contrasts, *P* < 0.05), there was no difference between the two lines within any treatment. Figure was created using Sigma Plot (SigmaPlot for Windows version 14.0, Copyright 2017, Systat Software, Inc. Palo Alto, CA, USA).

To test the effects of low-oxygen environments on the performance of male *A. suspensa*, pharate adults ~ 2 days prior to emergence were conditioned for 1 h with either hypoxia (7.3 ± 1.2 kPa of O_2_, 4.5 ± 0.8 kPa of CO_2_) or severe hypoxia (0.4 ± 0.1 kPa of O_2_, 0.8 ± 0.2 kPa of CO_2_). When these conditioned adults were sexually mature (6–8 days after adult emergence), total antioxidant capacity was elevated in both WT and transgenic lines compared to flies kept in normoxia (Table S1, *P*_atm_ = 0.0008), but the two lines did not differ in their responses (Table S1, *P*_line_ = 0.690). For sexually mature males that were not irradiated as pharate adults, severe hypoxia treatment significantly increased antioxidant capacity almost two-fold compared to insects kept in either normoxia or hypoxia (Fig. 1). Between non-irradiated males from normoxia and hypoxia treatments, however, no significant change in antioxidant capacity was observed (Fig. 1). The effect of radiation (rad) on total antioxidant capacity depended on atmospheric treatment (atm), as indicated by the significant interaction between these two fixed effects (Table S1, *P*_rad × atm_ =  < 0.0001). Males irradiated under hypoxia and non-irradiated males treated with severe hypoxia as pharate adults showed levels of total antioxidants nearly two-fold higher compared to normoxia non-irradiated males (Fig. 1). Males irradiated under either normoxia or severe-hypoxia and non-irradiated males treated with hypoxia as pharate adults did not differ significantly in levels of antioxidants relative to normoxia non-irradiated males (Fig. 1).

### Quality-control parameters

**Figure 2:**
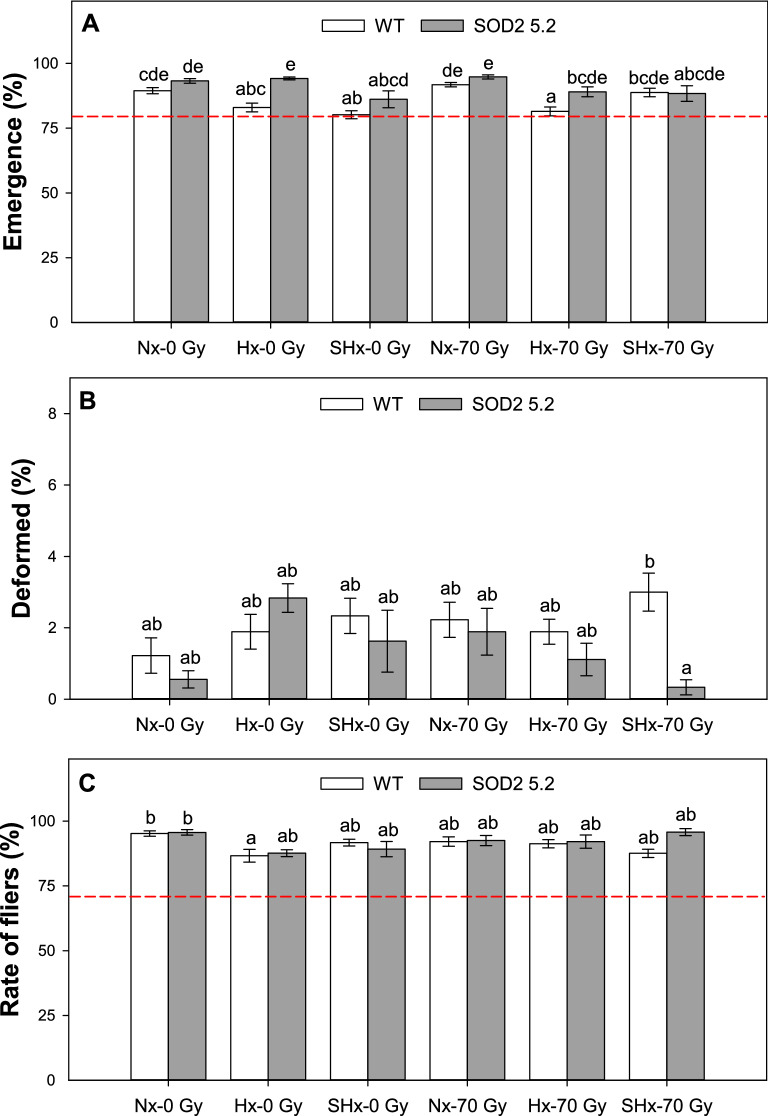
Quality control parameters assessed for non-irradiated and irradiated WT and SOD2 5.2 insects kept in normoxia (Nx) or treated with hypoxia (Hx) or severe hypoxia (SHx) conditioning for 1 h in the pharate adult stage. Percentage of adult emergence (**A**), deformed insects (**B**), and rate of fliers (**C**) were measured across treatments tested for each line. Graph bars represent means with standard errors for each treatment tested for both lines. Dashed red lines indicate 80% adult emergence in (**A**) and 70% fliers in (**C**), which are the minimum post-irradiation percentages acceptable for *Anastrepha suspensa* produced in SIT programs (FAO/IAEA/USDA 2019^29^). Bars followed by different letters are significantly different from each other (LS-means contrasts, *P* < 0.05). Figures were created using Sigma Plot (SigmaPlot for Windows version 14.0, Copyright 2017, Systat Software, Inc. Palo Alto, CA, USA).

For unirradiated males, adult emergence following hypoxic and severe-hypoxic conditioning of pharate adults decreased more markedly in WT than in SOD2 5.2 insects (Table S2-A, *P*_line_ =  < 0.0001, *P*_atm_ =  < 0.0001, *P*_line × atm_ = 0.0060), indicating that SOD2 overexpression may protect pharate adults from damage incurred from low-oxygen exposure. Severe hypoxia and hypoxia significantly reduced the emergence of non-irradiated and irradiated WT flies compared to normoxia treatments, respectively, but SOD2 overexpression conferred a protective effect on emergence of transgenic insects treated with hypoxia and trended in the same direction for the severe hypoxia treatment (Fig. 2A). For instance, the detrimental effect of severe-hypoxic conditioning on emergence of the non-irradiated insects was significant in WT flies and not significant in SOD2 5.2 insects (Fig. 2A), again suggesting that SOD2 overexpression confers protection against the oxidative damage associated with hypoxia-reperfusion responses. There was also a trend towards the SOD2 overexpression line having higher emergence than WT flies after exposure to severe hypoxia in the absence of radiation, but this difference was not statistically significant (Fig. 2A). Radiation treatment in the late pupal/pharate adult stage did not directly affect adult emergence.

There was a significant effect of line (Table S2-B, *P*_line_ = 0.0220) and radiation × atmosphere interaction (Table S2-B, Fig. 2B, *P*_rad × atm_ = 0.0100) on adult deformation, likely driven by the difference in deformation of flies irradiated in severe hypoxia—3% in WT and just 0.33% in SOD2 5.2. Radiation did not induce adult deformation across all treatments. These results suggest protective effects of SOD2 overexpression on responses to severe hypoxia late in pharate-adult development.

Flight ability of non-irradiated WT insects treated with hypoxia was significantly reduced compared to non-irradiated WT insects kept in normoxia (Fig. 2C, Table S2-C, *P*_atm_ =  < 0.0050). Despite a trend towards reduction in flight ability for irradiated WT flies, severe hypoxia did not significantly reduce the flight ability of WT insects relative to normoxia-treated insects (Fig. 2C). For unirradiated SOD2 insects there was no effect of either hypoxia or severe hypoxia on the rate of fliers, again suggesting that SOD2 overexpression had some protective effects on male quality. Radiation itself did not reduce the number of fliers in any of the treatment combinations.

### Sexual competitiveness

**Figure 3:**
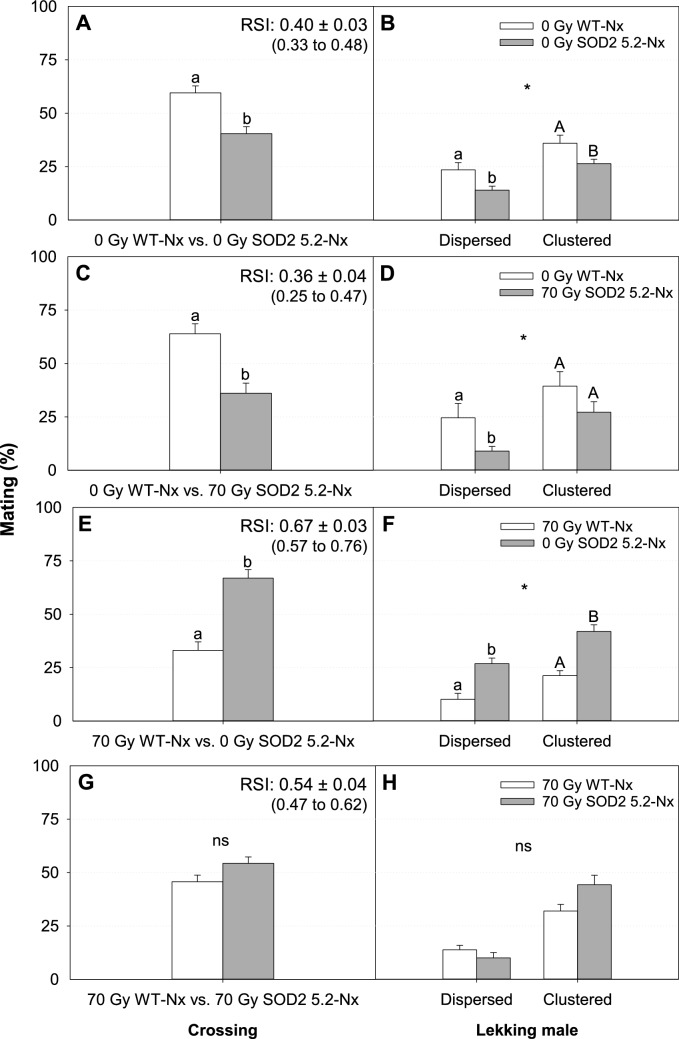
Mean mating success (SE) and positioning of lekking *Anastrepha suspensa* males treated under normoxia (Nx) in sexual competitiveness tests with a low male: female ratio (50 males: 25 females). (**A**) Proportion of matings obtained and (**B**) distribution of dispersed and clustered males within leks from matings achieved by non-irradiated WT (0 Gy WT-Nx) and SOD2 5.2 (0 Gy SOD2 5.2-Nx) males. (**C**) Proportion of matings obtained by and (**D**) distribution of dispersed and clustered males within leks from matings achieved by 0 Gy WT-Nx and irradiated SOD2 5.2 (70 Gy SOD2 5.2-Nx) males competing for the same females. (**E**) Proportion of matings obtained by and (**F**) distribution of dispersed and clustered males within leks from crossings between irradiated WT (70 Gy WT-Nx) and 0 Gy SOD2 5.2-Nx males competing for the same females. (**G**) Proportion of matings obtained by and H) distribution of dispersed and clustered males within leks from matings achieved by 70 Gy WT-Nx and 70 Gy SOD2 5.2-Nx males competing for the same females. *Statistical significance in our MANOVA models (Pillai's trace, *P* < 0.05); ‘ns’ means no statistically significant difference within each group (*P* > 0.05). Bars followed by different letters are significantly different (*P* < 0.05). Relative Sterility Indices (RSI) and their confidence intervals (parentheses) are shown for each crossing (**A**, **C**, **E**, **G**). RSI = (♂SOD2 5.2 × ♀WT)/(♂SOD2 5.2 × ♀WT) + (♂WT × ♀WT). Figures were created using Sigma Plot (SigmaPlot for Windows version 14.0, Copyright 2017, Systat Software, Inc. Palo Alto, CA, USA).

**Figure 4:**
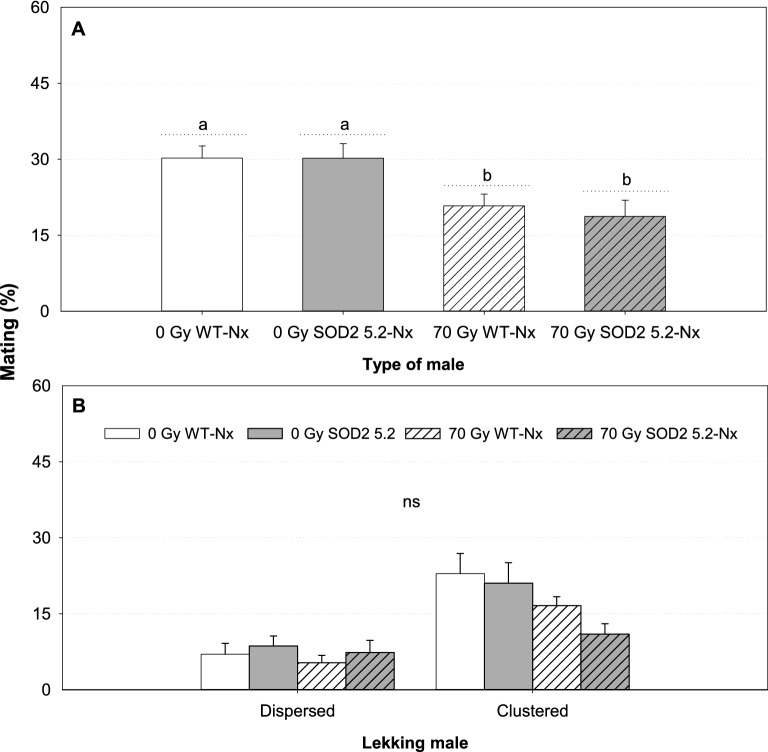
Mean mating success (SE) and position within leks of non-irradiated and normoxia-irradiated *Anastrepha suspensa* males competing together for the same females in tests with a high male: female ratio (100 males: 25 females). (**A**) Proportion of matings obtained by non-irradiated WT (0 Gy WT-Nx), non-irradiated SOD2 5.2 (0 Gy SOD2 5.2-Nx), irradiated WT (70 Gy WT-Nx), and irradiated SOD2 5.2 (70 Gy SOD2 5.2-Nx) males. (**B**) Distribution of dispersed and clustered males within leks from matings achieved by 0 Gy WT-Nx, 0 Gy SOD2 5.2-Nx, 70 Gy WT-Nx, and 70 Gy SOD2 5.2-Nx individuals. Bars followed by different letters are significantly different from each other (GLMM, LS-means contrasts, *P* < 0.05). Graph marked with ‘ns’ indicates no statistically significant difference within each group (dispersed or clustered) for our MANOVA model (*P* > 0.05). Figures were created using Sigma Plot (SigmaPlot for Windows version 14.0, Copyright 2017, Systat Software, Inc. Palo Alto, CA, USA).

The sexual competitiveness of WT and SOD2 5.2 males was evaluated at either a low (2:1) or a high (4:1) male: female ratio with combinations of non-irradiated or normoxia-irradiated males. Although there was a trend towards increased mating success of transgenic males compared to WT males when they were both irradiated, the mating competitiveness of WT-Nx 70 Gy and SOD2 5.2-Nx 70 Gy were not statistically distinguishable in field cages at 2:1 male: female ratio (Table S3, Fig. 3G, P = 0.0730, RSI of 0.54 ± 0.04). Yet, non-irradiated WT males were clearly more competitive than non-irradiated SOD2 5.2 males at a 2:1 male: female ratio (Table S3, Fig. 3A, P = 0.007, RSI 0.40 ± 0.03), strongly suggesting a negative side effect of transgenesis in the absence of oxidative stress. Interestingly, while SOD2 overexpression is detrimental in unirradiated males, the benefits of SOD2 overexpression become apparent after irradiation when SOD2 males no longer underperform compared to WT males. Thus, we believe that SOD2 overexpression could be beneficial if delivered in another construct or genomic position that is less costly to the performance of unstressed flies. As expected, irradiating insects under normoxia reduces sexual competitiveness compared to non-irradiated males in field cages with low male: female ratio (Fig. 3C, E), and high male: female ratio (Fig. 4A), regardless of their genetic background (Tables S3 and S4). Copulation duration (CD) and copulation latency (CL) were not different across the mating combinations with normoxia-treated males, with the exception of the test between WT-Nx 0 Gy versus SOD2 5.2-Nx 70 Gy, in which non-irradiated WT males mated faster (Table S5, CL: W = 3885.50, df = 1, *P* < 0.0032) and longer (CD: Table S5, F = 9.31, df = 1, *P* = 0.0027) than irradiated transgenic males.

**Figure 5:**
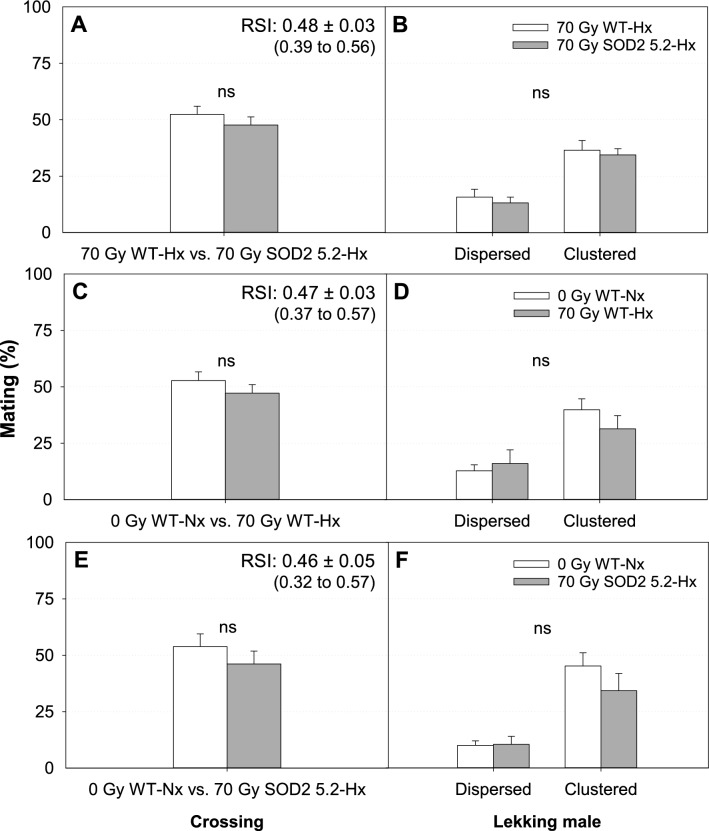
Mean mating success (SE) and positioning of lekking *Anastrepha suspensa* males treated under hypoxia (Hx) in sexual competitiveness tests with a low male: female ratio (50 males: 25 females). (**A**) Proportion of matings with WT females achieved by irradiated WT (70 Gy WT-Hx) and irradiated SOD2 5.2 (70 Gy SOD2 5.2-Hx) males. (**B**) Distribution of dispersed and clustered males within leks from matings achieved by 70 Gy WT-Hx and 70 Gy SOD2 5.2-Hx individuals. (**C**) Proportion of matings obtained by non-irradiated WT (0 Gy WT-Nx) and 70 Gy WT-Hx males competing for the same females. (**D**) Distribution of dispersed and clustered males within leks from matings achieved by 0 Gy WT-Nx and 70 Gy WT-Hx individuals. (**E**) Proportion of matings obtained by 0 Gy WT-Nx and 70 Gy SOD2 5.2-Hx males competing for the same WT females. (**F**) Distribution of dispersed and clustered males within leks from matings achieved by 0 Gy WT-Nx and 70 Gy SOD2 5.2-Hx individuals. Graphs marked with ‘ns’ indicates no statistically significant difference for either logistic regression or MANOVA models (*P* > 0.05). RSI = (♂SOD2 5.2 × ♀WT)/(♂SOD2 × 5.2 ♀WT) + (♂WT × ♀WT). Figures were created using Sigma Plot (SigmaPlot for Windows version 14.0, Copyright 2017, Systat Software, Inc. Palo Alto, CA, USA).

**Figure 6:**
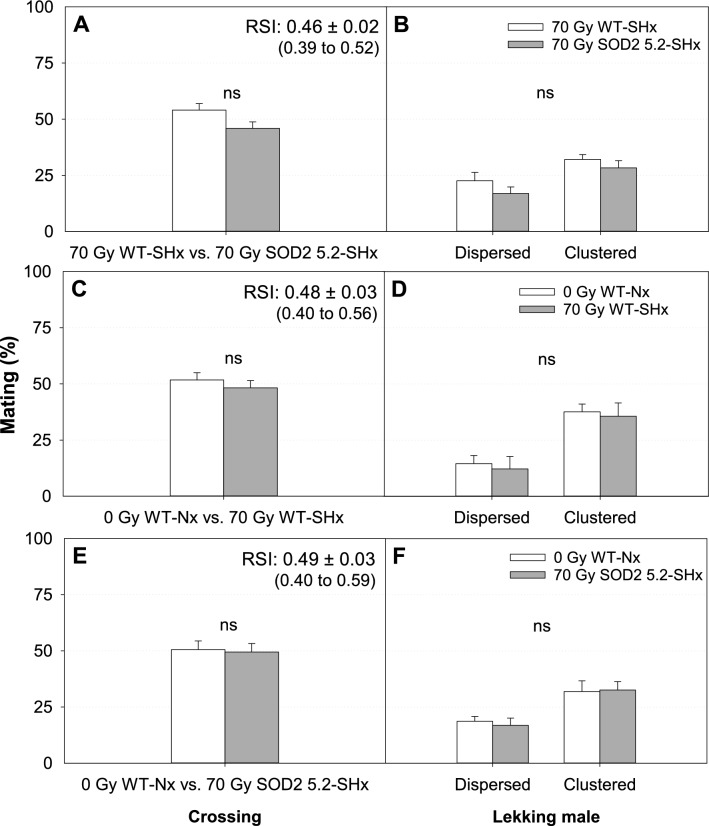
Mean mating success (SE) and positioning of lekking *Anastrepha suspensa* males treated under severe hypoxia (SHx) in sexual competitiveness tests with low male: female ratio (50 males: 25 females). (**A**) Proportion of matings with WT females achieved by irradiated WT (70 Gy WT-SHx) and irradiated SOD2 5.2 (70 Gy SOD2 5.2-SHx) males. (**B**) Distribution of dispersed and clustered males within leks from matings achieved by 70 Gy WT-SHx and 70 Gy SOD2 5.2-SHx individuals. (**C**) Proportion of matings obtained by non-irradiated WT (0 Gy WT-Nx) and 70 Gy WT-SHx males competing for the same females. (**D**) Distribution of dispersed and clustered males within leks from matings achieved by 0 Gy WT-Nx and 70 Gy WT-SHx individuals. (**E**) Proportion of matings obtained by 0 Gy WT-Nx and 70 Gy SOD2 5.2-SHx males competing for the same WT females. (**F**) Distribution of dispersed and clustered males within leks from matings achieved by 0 Gy WT-Nx and 70 Gy SOD2 5.2-SHx individuals. Graphs marked with ‘ns’ indicates no statistically significant difference for either logistic regression or MANOVA models (*P* > 0.05). RSI = (♂SOD2 5.2 × ♀WT)/(♂SOD2 5.2 × ♀WT) + (♂WT × ♀WT). Figures were created using Sigma Plot (SigmaPlot for Windows version 14.0, Copyright 2017, Systat Software, Inc. Palo Alto, CA, USA).

Sexual competitiveness of WT and SOD2 males irradiated under low-oxygen conditioning was also evaluated across all possible pairwise combinations, but only at a low (2:1) male: female ratio. Hypoxia (Fig. 5) and severe hypoxia (Fig. 6) treatments similarly improved the mating success of irradiated males by making them as competitive as non-irradiated WT males. Contrary to our hypothesis, overexpression of SOD2 did not interact synergistically with hypoxic or severe-hypoxic conditioning to further improve the mating success of *A. suspensa* males (Figs. 5A, 6A, Table S3). Overall, treating pharate adults for 1 h under hypoxic and severe-hypoxic conditions before irradiation was sufficient to make both WT and SOD2 5.2 males equally competitive to non-irradiated WT males (Table S3), as shown by their relative sterility index (RSI) values (Figs. 5, 6). Copulation duration and latency to copulation for cages involving hypoxia and severe hypoxia were not significantly different across any of the mating comparisons (Table S5).

### Distribution of successful males within the tree canopy

The position of each couple collected in the sexual competitiveness tests was recorded based on division of the tree canopy into 24 sections following the three-dimensional arrangement shown in Fig. S1. By taking into consideration the position in which the couples were collected, males were scored as dispersed if they mated in regions with just one copulation, or clustered if they mated in regions with two or more copulations. Subsequently, the proportion of copulations by dispersed versus clustered males was compared between lines in each atmospheric treatment × radiation exposure combination.

WT males were more successful in achieving copulations than SOD2 5.2 males when neither was irradiated nor exposed to atmospheric treatment, and this pattern held for both dispersed and clustered individuals (Fig. 3B, MANOVA, F_2,11(line)_ = 5.80, *P*_(line)_ = 0.0191; F_4,24(block)_ = 1.33, *P*_(block)_ = 0.2863). Similarly, and regardless of line, successful non-irradiated normoxia-treated males mated more frequently in regions with low frequencies of matings than normoxia irradiated males (Fig. 3F, WT-Nx 70 Gy vs. SOD2 5.2-Nx 0 Gy: MANOVA, F_2,10(line)_ = 16.81, *P*_(line)_ = 0.0039; F_6,22(block)_ = 0.84, *P*_(block)_ = 0.5553; and Fig. 3D, WT-Nx 0 Gy vs. SOD2 5.2-Nx 70 Gy: F_2,11(line)_ = 8.30, *P*_(line)_ = 0.0063; F_4,24(block)_ = 2.87, *P*_(block)_ = 0.0446).

Radiation treatment had contrasting effects on distribution of WT and SOD2 5.2 males within the tree canopy. The proportion of copulations by clustered individuals was lower for normoxia-irradiated WT males (Fig. 3F) than for normoxia-irradiated SOD2 5.2 males (Fig. 3D) relative to their non-irradiated counterparts. That is, normoxia-irradiated SOD2 5.2 males could afford to join leks in regions with potentially more male-male competition (indicated by the high-frequency of matings) than normoxia-irradiated WT males. However, no differences were found in the distribution of successful normoxia-irradiated WT and SOD2 5.2 males when competing directly in leks (Fig. 3H, WT-Nx 70 Gy vs. SOD2 5.2-Nx 70 Gy: MANOVA, F_2,9(line)_ = 1.58, *P*_(line)_ = 0.2575; F_8,20(block)_ = 1.23, *P*_(block)_ = 0.3316).

In cages with high (4:1) male: female ratio, the distribution of successful males did not differ among treatments. While higher numbers of non-irradiated males were found in highly competitive sectors compared to normoxia-irradiated males in tests with low male: female ratio, these differences fade out when sexual competitiveness tests were performed under high male: female ratio (100 males: 25 females) (Fig. 4B, WT-Nx 0 Gy vs. SOD2 5.2-Nx 0 Gy vs. WT-Nx 70 Gy vs. SOD2 5.2-Nx 70 Gy: MANOVA, F_6,44(line)_ = 1.79, *P*_(line)_ = 0.1236; F_4,44(block)_ = 1.11, *P*_(block)_ = 0.3630).

Hypoxic and severe hypoxic treatments before and during irradiation were beneficial to both WT and SOD2 5.2 males. Most copulations occurred between females and clustered males in tests with either non-irradiated males or individuals irradiated under hypoxic (Fig. 5) and severe-hypoxic (Fig. 6) conditions. There were no differences in the distribution of successful males in comparisons between WT and SOD2 5.2 males irradiated under hypoxia or severe hypoxia (MANOVA, WT-Hx 70 Gy vs. SOD2 5.2-Hx 70 Gy: F_2,12(line)_ = 0.47, *P*_(line)_ = 0.6386; F_6,26(block)_ = 2.25, *P*_(block)_ = 0.0699; WT-SHx 70 Gy vs. SOD2 5.2-SHx 70 Gy: F_2,12(line)_ = 1.07, *P*_(line)_ = 0.3729; F_6,26(block)_ = 0.75, *P*_(block)_ = 0.6182). Additionally, the positioning of successful males within the leks was similar in comparisons between WT or SOD2 5.2 males irradiated in hypoxia or severe hypoxia and non-irradiated WT males (MANOVA, WT-Nx 0 Gy vs. WT-Hx 70 Gy: F_2,7(line)_ = 0.24, *P*_(line)_ = 0.7931; F_4,16(block)_ = 0.38, *P*_(block)_ = 0.8207; WT-Nx 0 Gy vs. SOD2 5.2-Hx 70 Gy: F_2,5(line)_ = 0.43, *P*_(line)_ = 0.6722; F_4,12(block)_ = 0.72, *P*_(block)_ = 0.5933; WT-Nx 0 Gy vs. WT-SHx 70 Gy: F_2,7(line)_ = 0.31, *P*_(line)_ = 0.7450; F_4,16(block)_ = 0.89, *P*_(block)_ = 0.0.4934); WT-Nx 0 Gy vs. SOD2 5.2-SHx 70 Gy: F_2,7(line)_ = 0.15, *P*_(line)_ = 0.8647; F_4,16(block)_ = 0.67, *P*_(block)_ = 0.6191).

### Sterility

There was no effect of SOD2 overexpression on fertility. Irradiation of both WT and SOD2 males at 70 Gy in any atmospheric treatment prevented egg hatching: normoxia (WT-Nx 70 Gy: 2036 unhatched/ 0 hatched eggs, SOD2 5.2-Nx 70 Gy: 2241 unhatched/ 0 hatched eggs), hypoxia (WT-Hx 70 Gy: 2084 unhatched/ 0 hatched eggs, SOD2 5.2-Hx 70 Gy: 2073 unhatched/ 0 hatched eggs), and severe hypoxia (WT-SHx 70 Gy: 2488 unhatched/ 0 hatched eggs, SOD2 5.2-SHx 70 Gy: 2109 unhatched/ 0 hatched eggs). Controls obtained by crossing fertile WT females with non-irradiated WT and SOD2 5.2 males showed 90% ± 2% and 89% ± 1% egg viability, respectively.

## Discussion

Mitochondrial superoxide dismutase overexpression enhanced insect quality and modestly improved the mating success of irradiated transgenic males competing against irradiated WT counterparts in leks. Increased levels of SOD2 in transgenic males had limited protective effects on male quality related to hypoxia-reperfusion responses. Specifically, hypoxia-treated SOD2 males showed higher adult emergence rates compared to WT males, in both irradiated and unirradiated treatments. A lower proportion of deformities (partially emerged adults and wing damage) was observed in SOD2 males compared to WT males when irradiated in severe hypoxia, but not in other treatments.

We previously showed that SOD2 overexpression increased the mating success of normoxia-irradiated males up to 50% compared to irradiated WT males in small-scale mate choice tests^40^. In the sexual competitiveness tests under semi-natural field-cage conditions, the sexual advantage of SOD2 5.2 males over WT rivals observed in the laboratory was not as readily detectable with an increase in mating performance for irradiated SOD2 males of only 22% greater and a *P*-value that approached but did not reach our expected value of *P* < 0.05 for significance (*P* = 0.073 in Table S1). However, in our field-cage tests, normoxia-irradiated SOD2 5.2 males did mate more often in competitive lekking places where a higher frequency of mating events occurred than did normoxia-irradiated WT males, further suggesting that the transgenic insects might be more competitive than WT males in conditions where lekking occurs in the wild. We discuss four potential reasons to explain the inability to detect a difference in mating success in field cages when the improvement of SOD2 on mating was evident in small-cage trails^40^. The four factors are: (1) differences in optimal lek size, (2) context-dependent female mate choice, (3) irradiation dose rate, and (4) study statistical power.

First, although both small-scale and large-scale (field cages) mate choice tests were performed with a 2:1 male: female ratio, the total number of flies differed dramatically between the field cages (50 males, 25 females) and the small cages (2 males, 1 female), possibly diluting the sexual advantage of normoxia-irradiated transgenic males we observed in the small cages^40^. The larger groups of males and females in the field cages may favor the formation of large leks that, in turn, may have disrupted the ability of SOD2 5.2 males to monopolize both matings and access to females. Overall, male aggregation in leks benefits both high-ranking (attractive) and low-ranking (less attractive) males because it facilitates the access to females while reducing the risk of predation^42–44^. However, differences in optimal lek size between low-ranking and high-ranking males can limit mating success and the benefits of these aggregations. That is, attractive males obtain sexual advantages only in small leks, while unattractive males gain substantially in large leks^45,46^.

The contrast in optimal lek sizes between attractive and less attractive males drives the overcompensation approach adopted for SIT programs. For *C. capitata* inundative releases larger than 100 sterile males: 1 wild male are sometimes used in SIT programs to counterbalance the quality losses experienced by mass-reared and irradiated insects^27,47^. Because this overcompensation approach makes SIT relatively costly, alternative approaches that improve male quality instead of increasing release ratios have gained momentum in recent years^48,49^. Our previous work^40^ and this study both show that the use of transgenesis to increase enzymatic antioxidant activity may be used to enhance sterile male quality, but more work with additional lines that do not suffer detrimental effects of transgene insertion on mating competitiveness in the absence of radiation exposure are needed.

Second, the discrepancy between small cages^40^ and the field cages in this study could be a result of females in small arenas experiencing intense sexual harassment due to limited chances to escape the copulation attempts of insistent males, regardless of the male’s sexual quality. In a field cage, however, females have more opportunities to assess male condition without as much sexual harassment. Thus, mate choice tests in large field cages may provide better understanding of female preferences because they are more reflective of sexual selection in the field. In addition, female perception of male sexual signals can differ according to the environment^50,51^. *Anastrepha suspensa* males rely on chemical and acoustic displays to court females visiting leks^52^. Hence, it is reasonable to assume that female perceptions of acoustic vibration, wing beat frequency, pheromone composition and quality might differ if those signals were displayed in an enclosed small cage rather than in a semi-open field cage.

Third, it is possible that dissimilarities between this study and our previous work^40^ were due to differences in dose rates from different gamma irradiation sources used for each study. Insects in our previous small-scale mate choice tests were irradiated at a dose rate of ~ 8 Gy/min^40^, while males tested in field cages were irradiated at a dose rate of ~ 97 Gy/min. If radiation-induced oxidative damage is proportional to dose rate, then insects irradiated at lower dose rates will accumulate less oxidative damage in their cells. A few studies have assessed the effect of irradiation dose rate on fruit flies’ sterility and performance^53,54^. For instance, dose rates ranging from 5 to 80 Gy per minute affected neither fly quality nor sterility of *Bactrocera tryoni*^54^. However, *B. tryoni* individuals irradiated at high dose rates showed increased mortality under starvation compared to those irradiated at low dose rates^54^. Nonetheless, the effect of irradiation dose rates on sexual performance or oxidative stress have not been determined in tephritid fruit flies.

Last, our field-cage tests comparing the sexual competitiveness of normoxia-irradiated SOD2 and WT males were based on a relatively small sample size (n = 9). Therefore, this study had less statistical power to detect a modest difference in mating success between the WT and SOD2 lines than our previous study where we had many more replicates of small cages (~ 50). Post-hoc power analysis indicated that a sample size of 776 field cages would have 80% power to detect a small biological effect size consistent with a 10% difference in the mating success of normoxia-irradiated SOD2 5.2 males relative to their normoxia-irradiated WT counterparts, but these kinds of sample sizes are not possible given the labor-intensive work. Thus, we recognize the limitations of inferences we can make due to our small sample size.

Our findings corroborate the idea that endogenous antioxidant enzymes can play critical roles in the mechanisms of sexual selection in the face of severe oxidative stress conditions, such as irradiation^37^. Even though SOD2 activity was not measured in this study, our previous work showed that the mitochondrial superoxide dismutase activity of SOD2 5.2line males was 50% greater than WT males^40^. Superoxide dismutase (SOD) is a critical component of the antioxidant defenses of aerobic organisms^55,56^. Many vertebrates and invertebrates use antioxidant enzymes as a first line of protection against reactive oxygen species (ROS) and reactive nitrogen species (RNS) within cells^16,57,58^. Additionally, non-enzymatic antioxidants, such as glutathione and carotenoid pigments, can also play important direct and indirect roles in organismal antioxidative defense systems and should not be ignored^14,59^. More work on small-molecule antioxidants is needed in the context of sexual selection and male mating competitiveness in SIT.

The increase in total antioxidant activity we observed in response to anoxic conditioning in *A. suspensa* is not exclusive to our study. Previous correlative studies have shown that 1 h of anoxic conditioning of pharate adults (2 days before emergence) prior to irradiation (70 Gy) resulted in greater antioxidant capacity, specifically much higher SOD2 activity, less oxidative damage, better insect quality, and greater mating success than unconditioned irradiated males^37,38^. While we also observed an increase in sexual performance following low-oxygen conditioning, the conditioning treatment resulted in a reduction in several insect quality parameters, particularly emergence and percentage of fliers, contrary to what is reported in *C. capitata* under similar treatment conditions^60^. With respect to loss of quality, we cannot rule out a potential effect of hypercapnia (high [CO_2_]) on the reduction of adult emergence and flight ability in insects exposed to our hypoxic treatment (7.3 ± 1.2 kPa of O_2_, 4.5 ± 0.8 kPa of CO_2_). In *D. melanogaster*, exposure of adults to 100% CO_2_ for a duration as low as 15 min resulted in a long-lasting reduction in flight^61^. In tephritid fruit flies, the impact of short-term exposure (1 h) of pharate adults to mild concentrations of CO_2_ (≤ 5 kPa) on quality control parameters remains to be systematically evaluated. Usually, short-term exposure (~ 1 h) of fruit fly species used in operational SIT programs to hypoxia and hypercapnia does not result in detrimental effects^29^.

Contrary to our hypothesis, SOD2 overexpression did not interact synergistically with short-term low-oxygen conditioning hormesis to improve the mating success of transgenic males. Hypoxic or severe-hypoxic conditioning alone increased the total antioxidant capacity across all treatments relative to control (Nx-0 Gy) and similarly improved mating success in irradiated males from both lines (WT and SOD2 5.2) compared to non-irradiated rivals in leks. The lack of synergism between SOD2 overexpression and low oxygen treatments reported in our study may be explained based on the Preparation for Oxidative Stress (POS) framework. According to the POS theory, hypoxic conditioning has the potential to slightly increase ROS levels within cells, which, in turn, triggers an upregulation of antioxidant enzymes that prevent oxidative damage after reoxygenation^62–64^. That is, the high SOD2 levels in our transgenic line may have reduced the hormetic response usually triggered by the slight increase in ROS during hypoxia (as predicted by POS), and then minimized the upregulation of many antioxidant enzymes due to limited superoxide radicals.

Overall, increased antioxidant capacity during hypoxic or anoxic events has been observed for numerous organisms ranging from arthropods to diving seals and turtles, and increased antioxidant capacity is recognized as a common mechanism used to protect the organism from the stress of reoxygenation after hypoxia or anoxia exposure^65^. This study extends our earlier work on antioxidant protection in *A. suspensa*^37^ by directly comparing severe hypoxic (0.4 ± 0.1 kPa of O_2_, 0.8 ± 0.2 kPa of CO_2_) atmosphere to short-term hypoxic (7.3 ± 1.2 kPa of O_2_, 4.5 ± 0.8 kPa of CO_2_) conditioning treatments. Here males irradiated in both nitrogen and in a hypoxic atmosphere composed mainly of nitrogen with low levels of O_2_ and CO_2_ exhibited a similar protective effect to short-term anoxic conditioning. This finding is especially valuable, considering that SIT programs worldwide rely on natural oxygen depletion to safeguard sterile insect quality from the oxidative damage generated during irradiation, handling, and shipment procedures^30^.

In conclusion, we corroborate the previous findings of López-Martínez and Hahn^37,38^ and extend the same hormetic mechanism, first described for anoxic-conditioning, to less oxygen depleted hypoxic treatments. We also extend our earlier work on the benefits of SOD2 overexpression^40^ and show that while elevated SOD2 is beneficial, the benefits are not as dramatic in realistic field-cage settings. Alternative treatments focusing on the vast range of possibilities offered by enzymatic and non-enzymatic antioxidant protection to safeguard sterile insect quality and sexual competence should be extensively explored in future studies that seek to improve overall quality of irradiated insects used in SIT programs.

## Methods

### Insect strains and rearing protocol

Two lines of *A. suspensa* were used in our experiments. Transgenic mitochondrial SOD overexpression (SOD2 5.2), created from samples of Wild-Type (WT) colony from South Florida, express a Y-linked male-only insertion containing an extra copy of the *A. suspensa* SOD2 coding sequence and have SOD2 enzymatic activity ~ 50% higher than WT^40^. The rearing protocol used was based on a previous study^40^. Before the experiments and within 48 h after adult emergence, flies were sexed and placed in standard cages (29 cm long × 20 cm diameter) with unlimited access to water and artificial adult diet (3-parts sugar: 1-part yeast hydrolysate). Lines were maintained at 27 ± 1 °C, 50 ± 5% relative humidity, and 14L:10D photoperiod.

### Modified atmosphere and irradiation treatments

Three atmospheric regimes preceded irradiation treatment: normoxia (~ 20.9 kPa of O_2_, 0.04 kPa of CO_2_), hypoxia (7.3 ± 1.2 kPa of O_2_, 4.5 ± 0.8 kPa of CO_2_), and severe hypoxia (0.4 ± 0.1 kPa of O_2_, 0.8 ± 0.2 kPa of CO_2_). Normoxia (Nx) consisted of irradiating pupae, 2 days before emergence, in normal air. Hypoxia (Hx) treatment consisted of mechanically removing the air from a polypropylene bag containing a few hundred pupae and allowing the pupae to respire away the oxygen present and accumulate carbon dioxide, designed to approximate the process performed in SIT facilities. Severe hypoxia (SHx) was induced by flushing the bag containing a few hundred pupae with nitrogen for 1 min. Bags treated with low oxygen atmospheres were then sealed, placed into a second bag containing nitrogen that was also kept sealed for one hour before irradiation. Oxygen and carbon dioxide content was estimated using a CheckMate 3 gas analyzer (Dansensor, Denmark) with uncertainties of ± 0.01 (0–0.999% O_2_) or ± 1.0% (1–100% O_2_) for oxygen and ± 0.5% for carbon dioxide.

Two days prior to the expected time of emergence, WT and SOD2 5.2 pupae treated with a given atmospheric regime were irradiated with a target dose of 70 Gy^29^ using a Gammacell 220 (MDS Nordion, Ottawa, Canada) (dose rate of ~ 1.62 Gy s^−1^ over three months) located at the FAO/IAEA Insect Pest Control Laboratory, Seibersdorf, Austria. Non-irradiated pupae (control) were handled similarly. Dose accuracy was routinely verified using HD-V2 Gafchromic film (uncertainty 3.86% at 95% CI) placed either in a bag or a Petri dish at three different levels. HD-V2 films were read through an optical density meter (DoseReader 4, RadGen, Budapest, Hungary) 24 h after exposure.

### Total antioxidant capacity

Four sexually mature males (6–8 days old) from each treatment were collected into a 2-ml microcentrifuge tube, snap-frozen in liquid nitrogen, and stored at − 80 °C until biochemically assayed. Samples were homogenized using a FastPrep-24 homogenizer (MP Biomedicals, Santa Ana, CA, USA) with 2.8 mm ceramic beads (Bertin Technologies, France) in 500 µl of phosphate buffered saline (PBS) and centrifuged at 5000* g* for 5 min at 4 °C. The soluble protein concentration in the supernatant of each pool of four males was measured using a BCA kit (ThermoFisher, Rockford, IL, USA), and the concentration of each sample was adjusted to 2 µg/ml of soluble protein. The total antioxidant capacity of treated (Nx-70 Gy, Hx-0 Gy, Hx-70 Gy, SHx-0 Gy, and SHx-70 Gy) and untreated (Nx 0 Gy) WT and SOD2 5.2 males was determined using a total antioxidant colorimetric assay kit (Antioxidant Assay Kit, number 709001, Cayman Chemicals, Ann Arbor, MI, USA), according to the manufacturer's instructions. This assay measures the ability of antioxidants in the sample to inhibit the oxidation of ABTS (2,2′-Azino-di[3-ethylbenzthiazoline sulphonate]) to free radical cations (denoted as ABTS^+^) by metmyoglobin in the presence of hydrogen peroxide (441 µM). Antioxidant levels are indicated by the suppression of ABTS^+^ measured by reading the absorbance at 750 nm. The capacity of antioxidants in the samples to prevent ABTS oxidation was quantified using a standard curve of known concentrations of Trolox, a water-soluble tocopherol analog, and expressed as millimolar Trolox equivalent.

The difference in total antioxidant capacity of irradiated and non-irradiated WT and SOD2 5.2 males was analyzed using a general linear mixed model (GLMM) with type III sums of squares. Line (WT and SOD2 5.2), radiation (0 Gy and 70 Gy), low-oxygen atmosphere treatments (Nx, Hx, and SHx), and their interactions were modeled as fixed effects. Block, representing three temporal cohorts, was included as a random effect in the model. GLMM was performed using the *lme4* package^66^. Differences between treatments were determined using least-square means from *emmeans* package^67^. All data analyses in this study were performed using R^68^ (version 4.0.5) and R Studio^69^ (version 1.4.1106).

### Quality control parameters

Two days before emergence, 100 irradiated or non-irradiated pupae, treated with a low-oxygen atmosphere or not treated (normoxia), were placed inside a paper ring centered in the bottom of a darkened Petri dish (1.5 cm height × 7.7 cm diameter) surrounded by a black plexiglass tube (10 cm height × 8.9 cm diameter)^29^. The inner wall of the black tube was previously coated with a fine layer of talcum powder to prevent flies from crawling out instead of flying out. A resting area of 3 cm height was provided for newly emerged flies by wiping off the talcum powder from the inner wall of the tube. Emerged flies found outside the tubes were aspirated twice a day and scored as having successfully flown out of the tubes (fliers). Four days after the test set up, tubes were capped with a Petri dish lid. The non-fliers were then recorded, this includes the deformed flies (partially emerged or with damaged wings), normal emerged flies that failed to fly and non-emerged pupae^29^.

Proportion of emerged flies, deformation, and rate of fliers were analyzed using a GLMM with type III sums of squares. Line (WT and SOD2 5.2), radiation (0 Gy and 70 Gy), low-oxygen atmosphere treatments (Nx, Hx, and SHx), and their interactions were modeled as fixed effects. Block, representing three temporal cohorts, was included as a random effect in the model. GLMM was performed using the *lme4* package^66^. Differences between treatments were determined using least-square means from *emmeans* package^67^.

### Sexual competitiveness

Male sexual competitiveness and positioning within leks were evaluated under semi-natural conditions in standard field cages (2.0 m height × 3.0 m diameter) containing a *Ficus* sp. plant (Fig. S1), to provide substrate for sexual interactions^29^. Eleven combinations between different treated and untreated males were evaluated (Table S3). The day before the experiments, males were marked with a small dot of water-based paint (Washable Tempera Gouache, Alba) of different colors on the thorax^29^. The next day, for cages with low male: female ratio, 50 marked males, 25 males from each treatment, were released into the field cage and then, after 30 min, 25 WT untreated females were also released. For cages with high male: female ratio, 100 marked males, 25 males from each treatment, were released 30 min before the release of 25 females. Flies were observed from 14:00 to 19:00 h. Mating pairs were gently collected in plastic tubes, and their exact locations were identified with a numbered sticker (< 0.5 cm) and recorded to further sector classification. Mating frequency, copulation duration, copulation latency (the time from female release until copulation was initiated), and the location of copulations for each mate pair were also recorded.

Sexual competitiveness between males was estimated using the Relative Sterility Index (RSI = [♂SOD2 5.2 × ♀WT]/([♂SOD2 5.2 × ♀WT] + [♂WT × ♀WT])), which calculates the relative frequency of mating for each strain^29^. RSI varies from 0 (100% X males; X is a given group of males) to 1 (100% Y males; Y is another group of males), where 0.5 means equal competitiveness of both males (50% X, 50% Y). Differences among treatments in RSI were assessed by comparing the 95% confidence intervals for each combination. At least three cohorts were used for each of the sexual competitiveness tests. Mating frequencies, copulation duration, and copulation latencies of successful males were analyzed using GLMM (*lme4* package^66^) or Mann–Whitney test. For GLMM models fitting the low male: female ratio data, line and time of measurement (block) were modeled as fixed and random effects, respectively. For the GLMM model fitting the high male: female ratio data, line, radiation, and their interaction were modeled as fixed factors and time of measurement (block) was modeled as random effect. Differences between treatments were determined using least-square means from *emmeans* package^67^.

### Distribution of successful males within the tree canopy

The distribution of successful males used in the sexual competitiveness tests was inferred based on the location of each mate pair collected in the tree^29^. Successful males represented males that mated with the WT females released in the field cages. Male location within the leks was classified into 24 sectors following a three-dimensional arrangement (Fig. S1)^29^. To account for the dynamic nature of *A. suspensa* leks^52^ and to make males’ positioning within leks comparable between the tested insects across replications, the positions of successful males from each field cage test were grouped into two categories: dispersed (for places with a single copulation) and clustered (for sectors with two or more copulations). The effects of line and temporal cohorts (fixed effects) on the distribution of dispersed and clustered males within leks were assessed using multivariate analysis of variance (MANOVA, package *car*^70^) with type III sums of squares. Significant MANOVAs (*P* < 0.05) were followed by univariate F-tests to determine differences between WT and SOD2 5.2 males within each group of lekking males, dispersed or clustered.

### Sterility

Irradiated WT or SOD2 5.2 males treated or not with a low-oxygen atmosphere were crossed with virgin fertile WT females to assess male sterility. Briefly, 25 males from a specific treatment and 25 virgin WT females were transferred into a cage containing food and water ad libitum. After 15 days, an oviposition screen was placed on top of the cage from which eggs were collected every other day for at least a week until 2000 eggs per replication had been collected. Eggs were then transferred to a wet filter paper in a Petri dish (1.5 cm height × 9 cm diameter). At least 5 days after collection, unhatched and hatched eggs were scored and recorded. Five replicates were performed for each treatment, but no eggs hatched in any replicate or treatment including irradiation, so data were not formally analyzed.

## Supplementary Information

Supplementary Information 1.

Supplementary Information 2.
