# Supplementary material for: Mitochondrial superoxide dismutase overexpression and low oxygen conditioning hormesis improve the performance of irradiated sterile males
Source: Sci Rep. 2021 Oct 12;11:20182. doi: 10.1038/s41598-021-99594-1 (PMC8511041; doi:10.1038/s41598-021-99594-1)
Supplement: Supplementary file 1 — Supplementary Information 1. [file 41598_2021_99594_MOESM1_ESM.docx]

**Mitochondrial superoxide dismutase overexpression and low oxygen conditioning hormesis improve the performance of irradiated sterile males**

Vanessa S. Dias^1,2*^, Carlos Cáceres^1^, Andrew G. Parker^1^, Rui Pereira^1^, Güler Demirbas-Uzel^1^, Adly M. M. Abd-Alla^1^, Nicholas M. Teets^2,3^, Marc F. Schetelig^4^, Alfred M. Handler^5^ & Daniel A. Hahn^2^

^1^Insect Pest Control Subprogramme, Joint Food and Agriculture Organization (FAO)/ International Atomic Energy Agency (IAEA) Programme of Nuclear Techniques in Food and Agriculture, Vienna, A-1400, Austria

^2^Department of Entomology and Nematology, University of Florida, Gainesville, 32611, USA

^3^Department of Entomology, University of Kentucky, Lexington, 40546, USA

^4^Department of Insect Biotechnology in Plant Protection, Justus-Liebig-University Gießen, Gießen, 35394, Germany

^5^USDA/ARS, Center for Medical, Agricultural and Veterinary Entomology, Gainesville, 32608, USA

*[V.Dias-de-Castro@iaea.org](mailto:V.Dias-de-Castro@iaea.org) / [vanessasidias@hotmail.com](mailto:vanessasidias@hotmail.com)

**Supplementary information**

**SI Figures:**


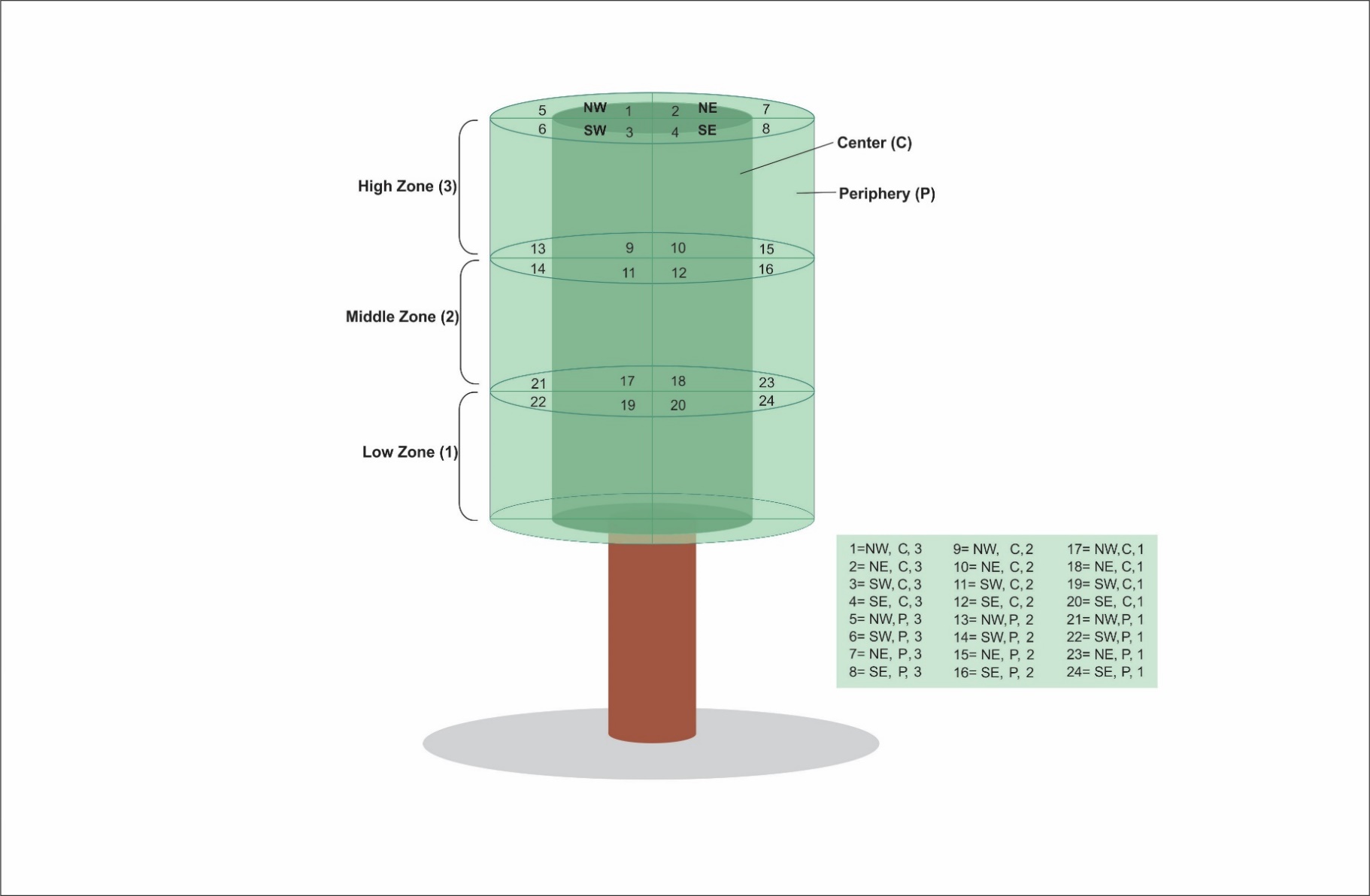


**Fig. S1.** Three-dimensional arrangement of the tree canopy used to classify the positioning of lekking *Anastrepha suspensa* males that mated in field cage tests. Males that mated were considered sexually successful. The location of each successful male within the leks was classified into 24 sectors, as follow: I) four cardinal quadrants, named as NE (northeast), NW (northwest), SE (southeast), and SW (southwest); II) three different height zones, numbered as 1 (bottom), 2 (middle), and 3 (top); III) two sectors describing depth in the tree canopy, represented as P (peripheral leaves situated approximately 5 cm from the edge of the canopy) and C (central leaves). Three different trees were used in the field cages tests. Thus, to account for the effect of tree configuration (e.g., foliage density and configuration) on male distribution, we categorize the distribution of the successful males in leks as dispersed (for males that mated in sectors with a single copulation) and clustered (for males that mated in sectors with two or more copulations).

**SI Tables:**

**Table S1.** Results of generalized linear mixed model (GLMM) testing for the total antioxidant capacity of non-irradiated and irradiated WT and SOD2 5.2 *Anastrepha suspensa* males under normoxia, hypoxia, and severe hypoxia. Fixed effects: line, atmosphere, radiation, and their interaction (×). Random effect = temporal cohort (block).

| Response | Predictors | d.f (n, d)^a^ | F | *P* |
| --- | --- | --- | --- | --- |
| Total antioxidant capacity | Line | 1, 51 | 0.16 | 0.6900 |
|  | Radiation | 1, 51 | 0.12 | 0.7400 |
|  | Atmosphere | 2, 51 | 8.23 | 0.0008^***^ |
|  | Line × Radiation | 1, 51 | 0.05 | 0.8200 |
|  | Line × Atmosphere | 2, 51 | 0.31 | 0.7300 |
|  | Radiation × Atmosphere | 2, 51 | 13.47 | <0.0001^***^ |
|  | Line × Radiation × Atmosphere | 2, 51 | 0.80 | 0.4500 |

^a^ Degrees of freedom (numerator, denominator)

^*^ Statistical significance (GLMM, *P* < 0.05)

**Table S2.** Results of GLMM testing for quality control parameters (emergence, deformation, rate of fliers) of WT and SOD2 5.2 males of *Anastrepha suspensa* treated or not with radiation under normoxia, hypoxia, and severe hypoxia. Fixed effects: line, atmosphere, radiation, and their interactions (×). Random effect = temporal cohort (block).

| Response | Predictors | d.f (n, d)^a^ | F | *P* |
| --- | --- | --- | --- | --- |
| (A) Emergence | Line | 1, 83 | 28.07 | <0.0001* |
|  | Radiation | 1, 83 | 1.82 | 0.1800 |
|  | Atmosphere | 2, 83 | 15.16 | <0.0001* |
|  | Line × Radiation | 1, 83 | 3.29 | 0.0700 |
|  | Line × Atmosphere | 2, 83 | 5.36 | 0.0060* |
|  | Radiation × Atmosphere | 2, 83 | 7.36 | 0.0010* |
|  | Line × Radiation × Atmosphere | 2, 83 | 0.55 | 0.5800 |
| (B) Deformation | Line | 1, 83 | 5.42 | 0.0220* |
|  | Radiation | 1, 83 | 0.0001 | 0.9900 |
|  | Atmosphere | 2, 83 | 0.89 | 0.4100 |
|  | Line × Radiation | 1, 83 | 3.43 | 0.0600 |
|  | Line × Atmosphere | 2, 83 | 2.74 | 0.0700 |
|  | Radiation × Atmosphere | 2, 83 | 4.37 | 0.0100* |
|  | Line × Radiation × Atmosphere | 2, 83 | 1.55 | 0.2200 |
| (C) Rate of fliers | Line | 1, 83 | 0.92 | 0.3400 |
|  | Radiation | 1, 83 | 0.60 | 0.1300 |
|  | Atmosphere | 2, 83 | 5.57 | <0.0050* |
|  | Line × Radiation | 1, 83 | 1.82 | 0.1800 |
|  | Line × Atmosphere | 2, 83 | 0.12 | 0.8900 |
|  | Radiation × Atmosphere | 2, 83 | 3.98 | 0.0200* |
|  | Line × Radiation × Atmosphere | 2, 83 | 2.86 | 0.0600 |
| ^a^ Degrees of freedom (numerator, denominator)  ^*^ Statistical significance (GLMM, *P* < 0.05) | | | | |

**Table S3.** GLMM testing for the effect of SOD2 overexpression, radiation and low oxygen treatments on male mating success of *Anastrepha suspensa*. Line and temporal cohort (block) were modeled as fixed and random effects, respectively.

| Crosses | n | | Line | | | | | | |
| --- | --- | --- | --- | --- | --- | --- | --- | --- | --- |
|  |  |  | df | | | F | *P* | | |
| *Normoxia* (Nx): air |  | |  | | |  |  | | |
| (1) 0 Gy WT-Nx vs 0 Gy SOD2 5.2-Nx^a^ | 8 | | 1 | | | 10.54 | 0.0070* | | |
| (2) 0 Gy WT-Nx vs 70 Gy SOD2 5.2-Nx^a^ | 8 | | 1 | | | 13.37 | 0.0040* | | |
| (3) 70 Gy WT-Nx vs 0 Gy SOD2 5.2-Nx^a^ | 8 | | 1 | | | 23.94 | 0.0004* | | |
| (4) 70 Gy WT-Nx vs 70 Gy SOD2 5.2-Nx^a^ | 8 | | 1 | | | 3.96 | 0.0730 | | |
| *Hypoxia* (Hx): *O*_2_= 7.3% (4.6 to 10%), CO_2_=4.5% (2.5 to 6.4%) | | | | | | | | |  |
| (5) 70 Gy WT-Hx vs 70 Gy SOD2 5.2-Hx^a^ | 9 | | 1 | | | 0.98 | 0.3400 | | |
| (6) 0 Gy WT-Nx vs 70 Gy WT-Hx^a^ | 6 | | 1 | | | 0.57 | 0.4730 | | |
| (7) 0 Gy WT-Nx vs 70 Gy SOD2 5.2-Hx^a^ | 6 | | 1 | | | 0.46 | 0.5150 | | |
| *Severe hypoxia* (SHx): *O*_2_= 0.4% (0.1 to 0.7%), CO_2_=0.8% (0.3 to 1.4%) | | | | | | | | |  |
| (8) 70 Gy WT-SHx vs 70 Gy SOD2 5.2-SHx^a^ | 9 | | 1 | | | 1.51 | 0.2410 | | |
| (9) 0 Gy WT-Nx vs 70 Gy WT-SHx^a^ | 6 | | 1 | | | 0.23 | 0.6352 | | |
| (10) 0 Gy WT-Nx vs 70 Gy SOD2 5.2-SHx^a^ | 6 | | 1 | | | 0.01 | 0.9050 | | |
| *Statistical significance (GLM, *P* < 0.05)  ^a^ Low (2:1) male: female ratio |  |  | |  |  | | |  |  |

**Table S4.** GLMM testing for the effect of SOD2 overexpression, radiation and low oxygen treatments on male mating success of *Anastrepha suspensa*. Line and temporal cohort (block) were modeled as fixed and random effects, respectively.

| Cross | Predictors | d.f (n, d) ^a^ | F | *P* |
| --- | --- | --- | --- | --- |
| 0 Gy WT-Nx vs 0 Gy SOD2 5.2-Nx vs 70 Gy WT-Nx vs 70 Gy SOD2 5.2-Nx^b^ | Line | 1, 22 | 0.0002 | 0.9886 |
|  | Radiation | 1, 22 | 8.9025 | 0.0068* |
|  | Line × Radiation | 1, 22 | 0.1408 | 0.7111 |

^a^ Degrees of freedom (numerator, denominator)

^b^ High (4:1) male: female ratio

^*^ Statistical significance (GLMM, *P* < 0.05)

**Table S5.** Proportion of mating, copulation latency, and copulation duration for WT and SOD2 males of *Anastrepha suspensa* competing in field cages, treated or not with radiation under normoxia, hypoxia, and severe hypoxia.

| Mating comparison | | PM^a^ ± SE | | Copulation parameters | | |
| --- | --- | --- | --- | --- | --- | --- |
|  |  |  |  | Male | CL^b^ ± SE (min) | CD^c^ ± SE (min) |
| *Normoxia* | |  | |  |  |  |
| (1) | 0 Gy WT vs  0 Gy SOD2 5.2 | 74% ± 4% | | 0 Gy WT | 36 ± 3 | 30 ± 1 |
|  |  |  |  | 0 Gy SOD | 31 ± 4 | 31 ± 2 |
| (2) | 0 Gy WT vs  70 Gy SOD2 5.2 | 81% ± 3% | | 0 Gy WT | 33 ± 3* | 31 ± 1** |
|  |  |  |  | 70 Gy SOD | 49 ± 5* | 25 ± 1** |
| (3) | 70 Gy WT vs  0 Gy SOD2 5.2 | 67% ± 4% | | 70 Gy WT | 65 ± 6 | 29 ± 1 |
|  |  |  |  | 0 Gy SOD | 61 ± 3 | 30 ± 1 |
| (4) | 70 Gy WT vs  70 Gy SOD2 5.2 | 82% ± 4% | | 70 Gy WT | 34 ± 3 | 29 ± 1 |
|  |  |  |  | 70 Gy SOD | 36 ± 3 | 31 ± 1 |
| (5) | 0 Gy WT vs | 85% ± 3% | | 0 Gy WT | 19 ± 3 | 32 ± 2 |
|  | 0 Gy SOD2 5.2 vs |  |  | 0 Gy SOD2 5.2 | 19 ± 2 | 33 ± 2 |
|  | 70 Gy SOD2 5.2 vs |  |  | 70 Gy WT | 19 ± 3 | 26 ± 2 |
|  | 70 Gy WT vs |  |  | 70 Gy SOD2 5.2 | 19 ± 3 | 27 ± 3 |
| *Hypoxia* | | | | | | |
| (6) | 70 Gy WT-Hx vs  70 Gy SOD2 5.2-Hx | 79% ± 3% | | 70 Gy WT-Hx | 27 ± 1 | 41 ± 3 |
|  |  |  |  | 70 Gy SOD2 5.2-Hx | 30 ± 2 | 38 ± 3 |
| (7) | 0 Gy- WT-Nx vs  70 Gy WT-Hx | 80% ± 5% | | 0 Gy WT-Nx | 30 ± 2 | 31 ± 3 |
|  |  |  |  | 70 Gy WT-Hx | 31 ± 2 | 35 ± 3 |
| (8) | 0 Gy WT-Nx vs  70 Gy SOD2 5.2-Hx | 65% ± 4% | | 0 Gy WT-Nx | 34 ± 2 | 38 ± 4 |
|  |  |  |  | 70 Gy SOD2 5.2-Hx | 35 ± 2 | 33 ± 4 |
| *Severe hypoxia* | | | | | | |
| (9) | 70 Gy WT-SHx vs  70 Gy SOD2 5.2-SHx | 75% ± 6% | | 70 Gy WT-SHx | 33 ± 2 | 31 ± 3 |
|  |  |  |  | 70 Gy SOD2 5.2-SHx | 29 ± 1 | 32 ± 3 |
| (10) | 0 Gy WT-Nx vs  70 Gy WT-SHx | 74% ± 5% | | 0 Gy WT-Nx | 34 ± 2 | 37 ± 4 |
|  |  |  |  | 70 Gy WT-SHx | 35 ± 2 | 40 ± 4 |
| (11) | 0 Gy WT-Nx vs  *7*0 Gy SOD2 5.2-SHx | 85% ± 4% | | 0 Gy WT-Nx | 32 ± 2 | 40 ± 4 |
|  |  |  |  | 70 Gy SOD2 5.2-SHx | 33 ± 2 | 39 ± 4 |
| ^a^ Proportion of mating | | |  | |  |  |
| ^b^ Copulation latency | | | | | | |
| *Statistical significance (Mann-Whitney test, W = 3885.5, *P* < 0.0032)  **Statistical significance (GLMM, F = 9.31, *P* = 0.0027) | | | | | | |
|  | | | | | | |
|  | | |  | |  |  |
